# Phytochrome B Mediates the Regulation of Chlorophyll Biosynthesis through Transcriptional Regulation of *ChlH* and *GUN4* in Rice Seedlings

**DOI:** 10.1371/journal.pone.0135408

**Published:** 2015-08-13

**Authors:** Noritoshi Inagaki, Keisuke Kinoshita, Takatoshi Kagawa, Ayumi Tanaka, Osamu Ueno, Hiroaki Shimada, Makoto Takano

**Affiliations:** 1 Photobiology and Photosynthesis Research Unit, National Institute of Agrobiological Sciences, Tsukuba, Ibaraki, Japan; 2 Functional Plant Research Unit, National Institute of Agrobiological Sciences, Tsukuba, Ibaraki, Japan; 3 Department of Biological Science and Technology, Tokyo University of Science, Katsushika, Tokyo, Japan; 4 Plant Adaptation Biology Group, Institute of Low Temperature Science, Hokkaido University, Sapporo, Hokkaido, Japan; 5 Faculty of Agriculture, Kyusyu University, Fukuoka, Fukuoka, Japan; 6 Genetically Modified Organism Research Center, National Institute of Agrobiological Sciences, Tsukuba, Ibaraki, Japan; National Taiwan University, TAIWAN

## Abstract

Accurate regulation of chlorophyll synthesis is crucial for chloroplast formation during the greening process in angiosperms. In this study, we examined the role of phytochrome B (phyB) in the regulation of chlorophyll synthesis in rice seedlings (*Oryza sativa* L.) through the characterization of a pale-green phenotype observed in the *phyB* mutant grown under continuous red light (Rc) irradiation. Our results show that the Rc-induced chlorophyll accumulation can be divided into two components—a phyB-dependent and a phyB-independent component, and that the pale-green phenotype is caused by the absence of the phyB-dependent component. To elucidate the role of the missing component we established an Rc-induced greening experiment, the results of which revealed that several genes encoding proteins on the chlorophyll branch were repressed in the *phyB* mutant. Notable among them were *ChlH* and *GUN4* genes, which encode subunit H and an activating factor of magnesium chelatase (Mg-chelatase), respectively, that were largely repressed in the mutant. Moreover, the kinetic profiles of chlorophyll precursors suggested that Mg-chelatase activity simultaneously decreased with the reduction in the transcript levels of *ChlH* and *GUN4*. These results suggest that phyB mediates the regulation of chlorophyll synthesis through transcriptional regulation of these two genes, whose products exert their action at the branching point of the chlorophyll biosynthesis pathway. Reduction of 5-aminolevulinic acid (5-ALA) synthesis could be detected in the mutant, but the kinetic profiles of chlorophyll precursors indicated that it was an event posterior to the reduction of the Mg-chelatase activity. It means that the repression of 5-ALA synthesis should not be a triggering event for the appearance of the pale-green phenotype. Instead, the repression of 5-ALA synthesis might be important for the subsequent stabilization of the pale-green phenotype for preventing excessive accumulation of hazardous chlorophyll precursors, which is an inevitable consequence of the reduction of Mg-chelatase activity.

## Introduction

Among the various environmental signals that plants utilize to optimize their growth, development and reproduction, light is the most important one [1]. To sense light signals, plants are equipped with a series of photoreceptors, such as phytochromes, cryptochromes, LOV (light, oxygen or voltage) domain containing blue light receptors and a UV-B receptor molecule, UV RESISTANCE LOCUS 8 (UVR8) protein [2–4]. Phytochrome was initially recognized to be a red/far-red light (R/FR) receptor, responsible for the germination of photoblastic seeds [5]. Now, it is established that phytochrome is a ubiquitous photoreceptor in plants that mediates various responses, such as photomorphogenesis, shade avoidance and photoperiodic control of flowering [6, 7]. Phytochromes have two reciprocally changeable forms, namely an R absorbing form (P_R_) and an FR absorbing form (P_FR_) [8]. Newly synthesized phytochromes are P_R_ and physiologically inactive. P_R_ converts to P_FR_ upon perception of R, and similarly, P_FR_ reverts to P_R_ after perception of FR. The reciprocal transformation between the two forms is critical to transduce light stimuli into biological signals [9]. Physiological studies revealed that the phytochrome-mediated responses could be classified into at least three modes, namely very low fluence response (VLFR), low fluence response (LFR) and high irradiance response (ex. R-HIR or FR-HIR) [10]. Among the three modes, the LFR displays a unique feature in that it can be cancelled by subsequent FR irradiation, which is based on the reciprocal transformation between P_FR_ and P_R_ [10]. Phytochrome genes form a small family. In rice (*Oryza sativa* L.), it consists of three genes, *PHYA*, *PHYB* and *PHYC* [6, 11–13]. We have isolated a series of rice mutants deficient in all the phytochrome genes [14, 15] and generated comprehensive combinations of double and triple mutants [15, 16]. These mutants have contributed to the elucidation of the function of phytochromes in rice plants [17–19].

Chlorophyll is the major photosynthetic pigment in plants. It is synthesized from a glutamyl-tRNA through a series of enzymatic steps (S1 Fig) that take place in the plastid [20, 21]. Up to protoporphyrin IX formation, chlorophyll and heme share the same biosynthesis pathway. The first specific step for chlorophyll synthesis is mediated by magnesium chelatase (Mg-chelatase), which introduces an Mg^2+^ ion into protoporphyrin IX to form Mg-protoporphyrin IX. The enzyme consists of three subunits, ChlH, ChlI and ChlD [22], but the full activation of the enzyme requires an additional protein, Genomes Uncoupled 4 (GUN4) [23].

When angiosperms seeds germinate in complete darkness, chlorophyll synthesis in the seedlings is prevented at various steps of the biosynthetic pathway. Activity of glutamyl-tRNA reductase is repressed by binding of the FLUORESCENT IN BLUE LIGHT (FLU) protein, which causes reduced synthesis of 5-aminolevulinic acid (5-ALA) [24, 25]. Expression of the majority of genes involved in chlorophyll synthesis is repressed in darkness [26, 27]. Chlorophyll synthesis is tightly blocked at the protochlorophyllide oxidoreductase (POR) reaction stage in darkness [28] and, as a consequence, plastids in dark-grown seedlings (etioplasts) contain no chlorophyll. Instead, they are filled with a crystalline structure, the prolamellar body, consisting mainly of complexes of protochlorophyllide and POR [29].

On the other hand, irradiation of the seedlings triggers rapid synthesis of chlorophyll along with the simultaneous development of chloroplast [30]. During the process, chlorophyll synthesis must be regulated delicately. Plants have developed several layers of transcriptional, translational and post-translational mechanisms to coordinate chloroplast development and chlorophyll synthesis [21]. Available evidence from physiological, genetic and molecular biological research work indicates that this regulation is mediated in part by phytochromes [27, 31–35], but the mechanism of this still remains to be elucidated.

In this study, we characterized a pale-green phenotype observed in rice *phyB* seedlings grown under continuous R (Rc) irradiation. We determined that phyB is indispensable for the robust induction of several genes encoding proteins acting on the chlorophyll branch upon Rc irradiation. In particular, we found that the expression of *ChlH* and *GUN4* genes, which was drastically induced by R in the wild type (WT) seedlings, was repressed in the *phyB* mutant. Chlorophyll precursor content profiles indicated that Mg-chelatase activity in the *phyB* mutant was reduced in parallel to the repression of the two genes, which should consequently lead to the appearance of the pale-green phenotype. These results can be interpreted that phyB signals raise Mg-chelatase activity through the transcription of the *ChlH* and *GUN4* genes in rice plants.

## Materials and Methods

### Plant materials and growth conditions

Rice (*Oryza sativa* L.) phytochrome mutants, *phyA-4*, *phyB-1*, *phyB-2*, *phyB-3*, *phyB-4*, *phyB-5*, *phyC-1*, *phyA-4 phyC-1* and *phyA-4 phyB-1* were used in this study [14, 15]. Genetic background of *phyA-4*, *phyB-1*, *phyB-2*, *phyC-1*, *phyA-4 phyC-1* and *phyA-4 phyB-1* was Nipponbare, while that of *phyB-3*, *phyB-4* and *phyB-5* was Norin-8. Husked seeds were surface-sterilized by soaking twice in hypochlorous acid solution for 15 min with vigorous shaking. Seeds were washed three times with sterile water and spread onto 0.4% (w/v) agar in water. Seedlings were grown at 28°C in darkness or under various light conditions described in the text or figure legends. We used an R-light emitting diode (LED) panel (Model LED-R, EYELA, http://www.eyelaworld.com/) and an FR-LED panel (Model LED-FR, EYELA) for monochromatic light sources. The FR-LED panel was mounted into a filter box with one layer of acryl filter (KYOWALITE PG SP-60-3K 202, KURARAY, http://www.kuraray.co.jp/en/).

### Chlorophyll extraction and measurement

Aerial parts of the seedlings were harvested, weighed and kept at −80°C in complete darkness until extraction of chlorophylls. Frozen samples were mechanically ground in a sampling tube with two stainless-steel beads (5 mm diameter) by a bead mill (TissueLyser II, QIAGEN, http://www.qiagen.com/). Cooled acetone was added to the resultant powder and chlorophylls were extracted from the samples with vigorous shaking. Resultant mixtures were centrifuged at 17,600×*g* for 10 min at 4°C and supernatants were stored in another sampling tube. Extraction was repeated three times from the samples. Acetone content of the pooled extracts was adjusted to 80% (v/v) by addition of water. Absorbance at 646.6, 663.6 and 750.0 nm of the extracts was measured with a spectrophotometer (UV-2400PC, Shimadzu, http://www.shimadzu.com/). Concentrations of chlorophyll *a* and *b* in the extracts were calculated from these values through the following equations reported by Porra et al. [36]:

$$\text{chlorophyll}\;a\;\left(\mu \text{g}\;{\text{ml}}^{-1}\right)=12.25\times \left(A_{663.6}-A_{750.0}\right)-2.55\times \left(A_{646.6}-A_{750.0}\right)$$

$$\text{chlorophyll}\;b\;\left(\mu \text{g}\;{\text{ml}}^{-1}\right)=20.31\times \left(A_{646.6}-A_{750.0}\right)-4.91\times \left(A_{663.6}-A_{750.0}\right)$$

### Transmission electron microscopic observation

Segments excised from leaf sheaths were fixed in 3% (v/v) glutaraldehyde in 50 mM sodium phosphate buffer (pH 6.8) at room temperature for 1.5 h, then washed with phosphate buffer and post-fixed in 2% (w/v) OsO_4_ in phosphate buffer for 2 h. Subsequently, they were dehydrated through an acetone series and embedded into Spurr’s Resin (Nisshin EM, Tokyo, Japan). The transverse ultrathin sections of the leaf sheaths were stained with lead citrate, and observed under a transmission electron microscope (TEM) (H-7100, Hitachi High-Technologies, http://www.hitachi-hightech.com/) at 75 kV.

### Western blotting analysis

Seedlings grown under Rc irradiation for 9 days were harvested and weighed. Aerial parts of the seedlings were ground in a mortar and pestle in 25 mM HEPES-NaOH buffer (pH 7.5) containing 20 mM NaCl, 6 mM MgCl_2_ and 1 mM EDTA. Proteins in the extracts were mixed with same volume of 2× SDS-PAGE loading buffer. Resultant extracts from 0.67 mg of fresh weight of samples were subjected to SDS-PAGE using gels containing 15% (w/v) acrylamide and 6 M urea. Separated proteins in the gels were blotted onto a nitrocellulose membrane (Optitran BA-S 85 Reinforced NC, Schleicher & Schuell, http://www.gelifesciences.com/) electrophoretically. The procedures for immunochemical detection were carried out as described elsewhere [15] using alkaline phosphatase-conjugated AffiniPure Goat Anti Rabbit IgG (H+L) (Jackson ImmunoResearch Laboratories, https://www.jacksonimmuno.com/) with BCIP/NBT (5-Bromo-4-chloro-3-indolylphosphate/Nitro-blue tetrazolium) Color Development Substrate (Bio-Rad, http://www.bio-rad.com/). A dilution series of the wild type sample was conducted to ensure that the evaluation of proteins was carried out within the limits of detection of the method and below the saturation levels of proteins.

### RNA extraction and estimation of transcript levels

Total RNA was isolated from aerial parts of the seedlings using RNeasy Plant Mini kit (QIAGEN). The RNA samples were treated with RNase-free DNase I (QIAGEN) according to the manufacturer’s instructions. Two μg of total RNA were reverse-transcribed using Superscript III reverse transcriptase (Invitrogen, http://www.lifetechnologies.com/) and oligo dT primers to generate cDNAs. One μl of cDNA solution was applied to PCR analysis using *Taq* DNA polymerase (QIAGEN). PCR products were separated by agarose gel electrophoresis and visualized by ethidium bromide staining. Gel images were obtained with the AlphaImager Mini System (ProteinSimple, http://www.proteinsimple.com/) and the relative intensities of signals were estimated from the gel images using ImageJ software (http://rsb.info.nih.gov/ij/). Three biological repetitions of RT-PCR assays were carried out to establish statistical reliability. Levels of *OsUBQ* (D12629) transcript were stable during our experimental condition; therefore, we used this transcript as the basal standard. Real-time PCR assays were carried out using a Thermal Cycler Dice Real Time System (TAKARA-Bio, http://www.takara-bio.com/) with QuantiTect SYBR Green PCR Kit (QIAGEN). Sequences of PCR primers used in this study are listed in S1 Table.

### Measurement of 5-ALA synthesis rate

Basic procedure for the determination of 5-ALA accumulation rate was similar to what has been described previously [25, 37]. Seedlings were chopped to make 5 mm segments and incubated for 90 min at 28°C on filter papers soaked in 50 mM potassium phosphate buffer (pH 6.8) containing 60 mM levulinic acid. After incubation, the segments were weighed, frozen in liquid nitrogen and stored at −80°C. Approximately 0.7 g of the frozen tissue was homogenized in 800μl of 50 mM potassium phosphate buffer (pH 6.8) by vigorous shaking with two tungsten carbide beads (diameter 5 mm) in a bead mill (TissueLyser II, QIAGEN). Resultant extracts were centrifuged at 17,610×*g* for 10 min at 4°C. After centrifugation, 600μl of the supernatant was condensated with 120μl of ethylacetoacetate by boiling for 10 min in a water bath. After cooling on ice, 720μl of the modified Ehrlich’s reagent (0.2 g *p*-dimethylamino benzaldehydo, 8.4 ml of acetic acid and 1.6 ml of 70% perchloric acid, freshly prepared each time) was added and incubated for 10 min at room temperature. The resultant solutions were centrifuged at 17,610×*g* for 5 min at 25°C and absorbance at 553 nm of the supernatant was measured with a spectrophotometer, UV-2400PC (Shimadzu). Content of the 5-ALA in the samples was calculated from calibration curves determined from solutions of commercial 5-ALA (Sigma-Aldrich, https://www.sigmaaldrich.com/).

### Measurement of chlorophyll precursor content

Basic procedures for the measurement of content of three chlorophyll precursors, protoporphyrin IX, Mg-protoporphyrin IX and Mg-protoporphyrin IX monomethylester were followed by an HPLC-based procedure as described previously [38]. Extraction of chlorophyll precursors from seedlings was carried out using the same procedure as for chlorophyll extraction described above. The acetone extracts were applied to a HPLC system (LC10A VP system, Shimadzu) equipped with a reverse phase column (Symmetry C8, 150 mm × 4.6 mm, 3.5μm particle size, Waters, http://www.waters.com/) with a guard column (C8, 4.0 mm × 3.0 mm, Phenomenex, http://www.phenomenex.com/). Chlorophyll precursors were eluted by an anomalous gradient from solvent A (methanol:acetonitrile: 0.25 M pyridine = 50:25:25) to solvent B (methanol:acetonitrile:acetone = 20:60:20). Eluents were monitored with a fluorescence detector (RF-550, Shimadzu). For protoporphyrin IX detection, the excitation wavelength was set at 400 nm and fluorescence emission was detected at 634 nm, while for detections of Mg-protoporphyrin IX and Mg-protoporphyrin IX monomethylester, the excitation wavelength was set at 417 nm and fluorescence emission was detected at 600 nm. Content of these precursors was estimated through calibration curves determined from standard substances (Frontier Scientific, http://www.frontiersci.com/).

## Results

### phyB deficiency causes a pale-green phenotype under R

**Fig 1:**
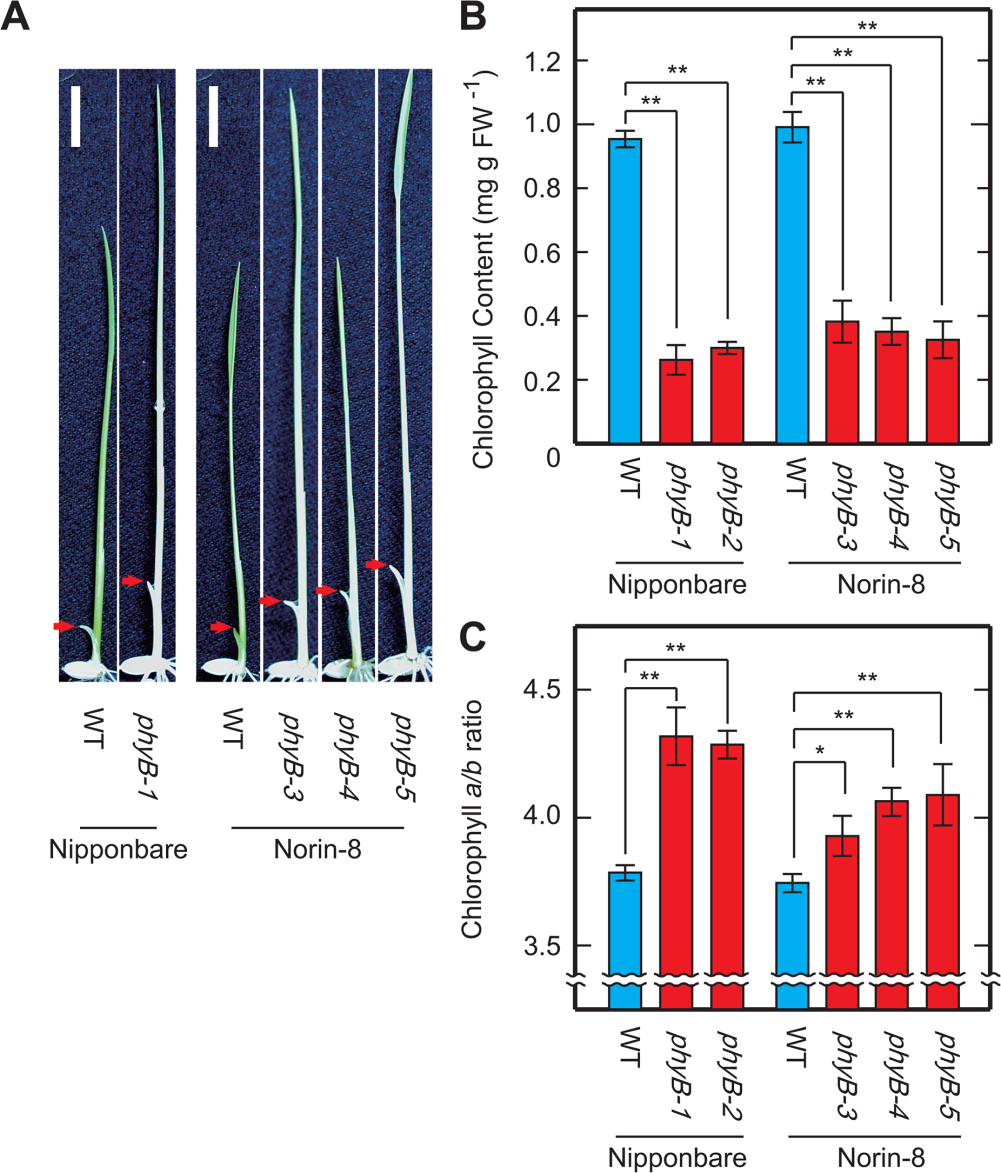
Phenotypes of the rice *phyB* mutants. (A) WT and *phyB* seedlings grown under 15μmol m^-2^ s^-1^ of Rc for 7 days. Arrows indicate tip of coleoptiles. Scale bars are 10 mm. Chlorophyll content (B) and chlorophyll *a*/*b* ratios (C) in aerial part of the WT and *phyB* seedlings grown under Rc for 9 days. Data are given as means calculated from three replicates and error bars represent their SD. Symbols, ** and * indicate statistically significant differences compared to WT at *P* < 0.01 and *P* < 0.05, respectively, calculated by Welch’s *t*-test.

**Fig 2:**
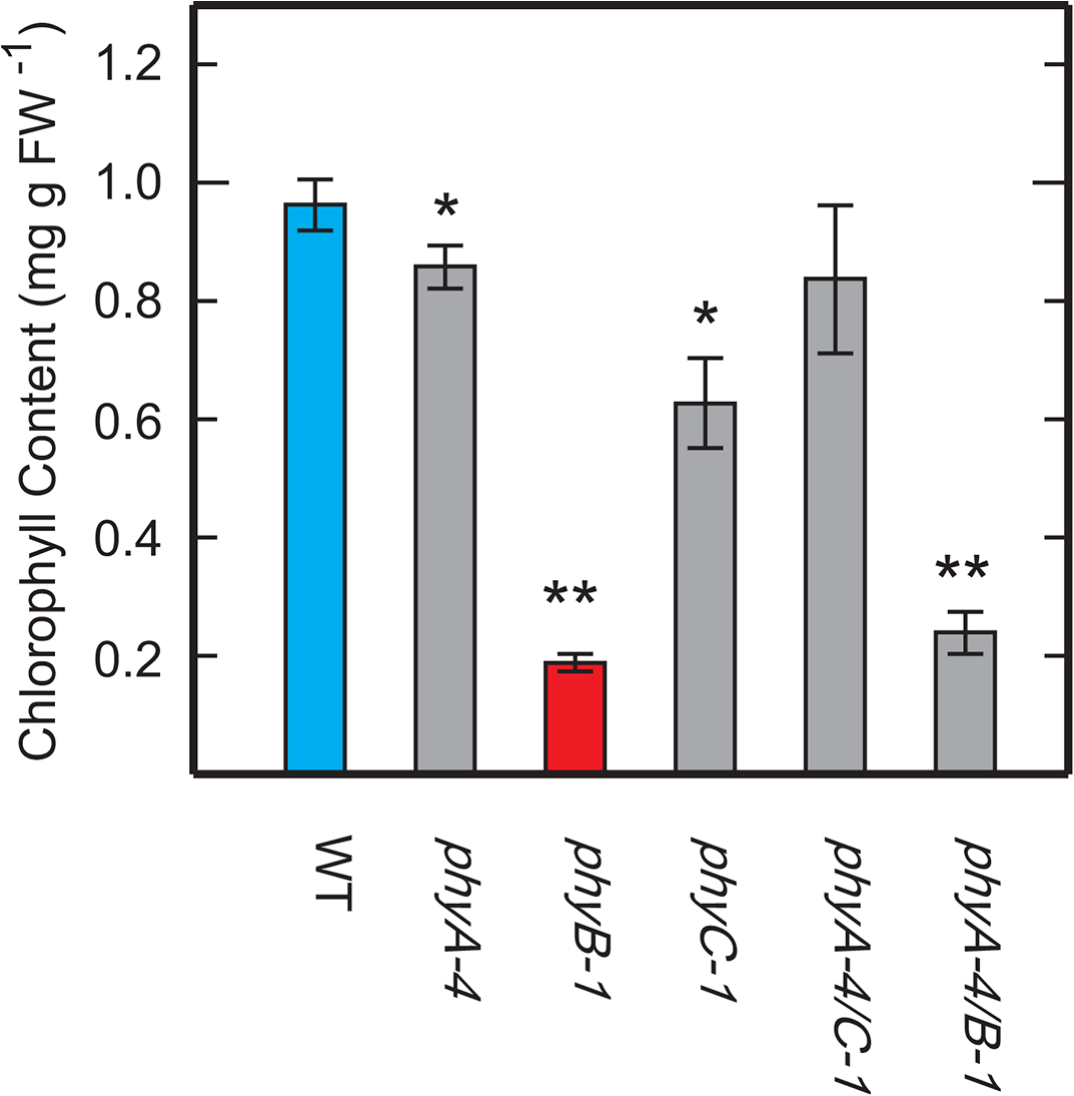
Chlorophyll content in aerial parts of the phytochrome mutants grown under Rc for 9 days. Each value represents the mean with SD of three replicates. Symbols, ** and * indicate statistically significant differences compared to WT at *P* < 0.01 and *P* < 0.05, respectively, calculated by Welch’s *t*-test.

We have previously isolated rice *phyB* mutants (*phyB-1* to *-5*) through a phenotypic screening [15] based on the finding that inhibition of coleoptile elongation by R is mediated by phytochromes [39]. The *phyB* mutants were identified from seedlings with a slightly elongated coleoptile (Fig 1A) when grown under Rc irradiation. We have also noticed that the *phyB* mutants displayed a pale-green feature under the screening conditions (Fig 1A). Chlorophyll content in the aerial parts of the *phyB* seedlings grown under Rc (15μmol m^-2^ s^-1^) for 9 days was in the 20–40% range compared to WT (Fig 1B), indicating that the pale-green phenotype was due to reduced chlorophyll content. Values of chlorophyll *a*/*b* ratio in the *phyB* seedlings were slightly higher than those of WT (Fig 1C). It should be noted that deficiencies of other phytochromes, such as, phyA and phyC, also lead to decreased chlorophyll content, but to a lesser extent than that of phyB deficiency (Fig 2).

### FR-enriched light treatment also causes pale-green phenotype

**Fig 3:**
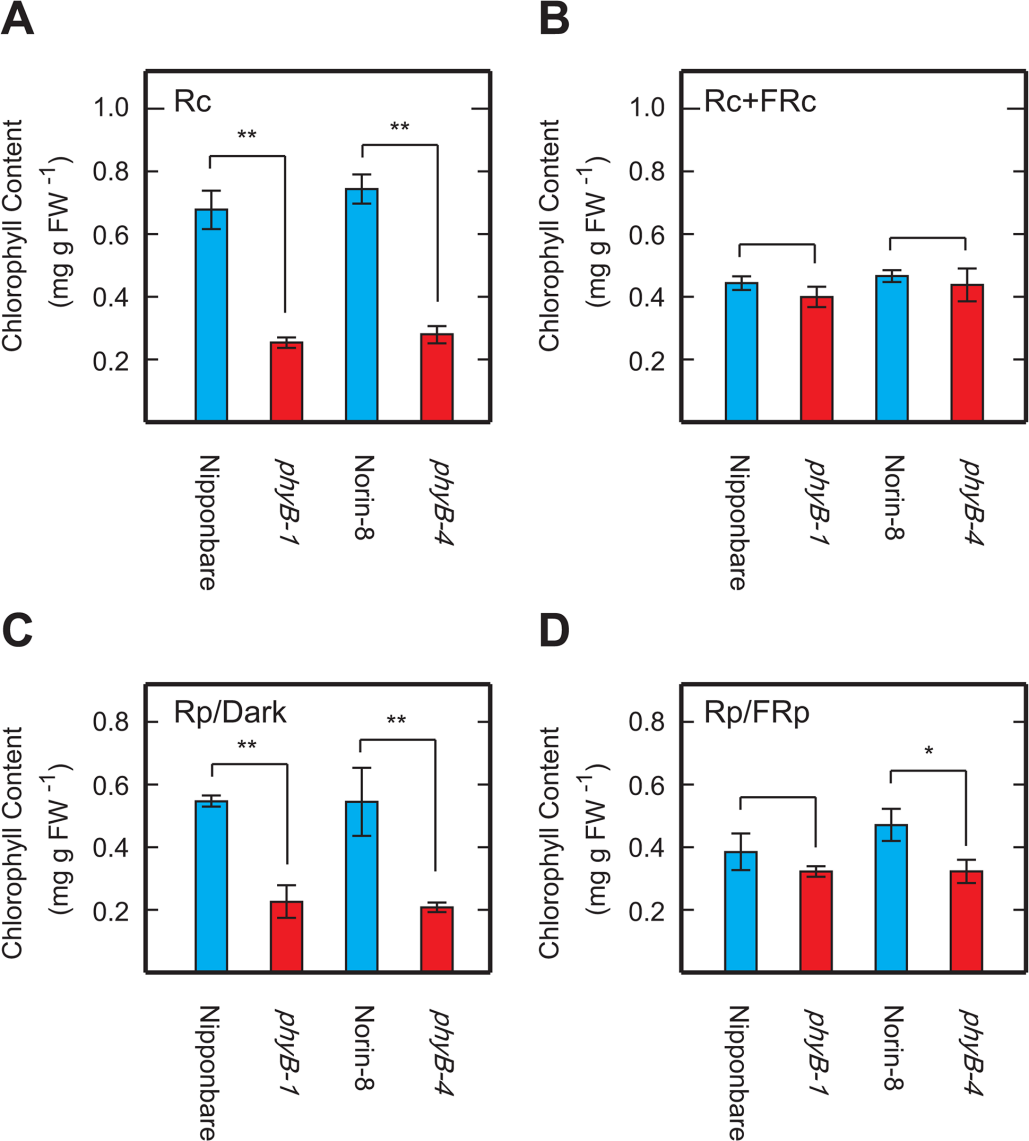
Effects of supplemental FR irradiations on chlorophyll content in WT seedlings. Chlorophyll content in aerial parts of seedlings grown under 5μmol m^-2^ s^-1^ of Rc without (A) or with (B) supplemental FRc (30μmol m^-2^ s^-1^) for 9 days. Chlorophyll content in seedlings grown under cycles consisting of 2 min of Rp (15μmol m^-2^ s^-1^) and 8 min of darkness (C) or 2 min of Rp and 8 min of FRp (15μmol m^-2^ s^-1^) (D). Each value represents the mean with SD of at least three replications. Symbols, ** and * indicate statistically significant differences compared to WT at a levels of *P* < 0.01 and *P* < 0.05, respectively, calculated by Welch’s *t*-test.

If the pale-green phenotype is due to phyB deficiency, it should be possible to mimic the phenotype in WT seedlings under conditions available to cancel LFRs. Chlorophyll content of WT seedlings grown under Rc (5μmol m^-2^ s^-1^) with or without simultaneous strong continuous FR (FRc) (30μmol m^-2^ s^-1^) was measured and compared with those of the *phyB* seedlings (Fig 3A and 3B). In spite of the fact that seedlings were exposed to enough R irradiation to accumulate chlorophylls (Fig 3A), simultaneous FRc irradiation cancelled the Rc effect and dropped the chlorophyll content of the WT seedlings to levels comparable with those of the *phyB* mutants (Fig 3B). In addition, chlorophyll accumulation induced by intermittent irradiation cycles consisting of a 2 min pulse of R (Rp) (15μmol m^-2^ s^-1^) and an 8 min of darkness in the WT seedlings (Fig 3C) was also cancelled by 8 min of FR pulses (FRp) (15μmol m^-2^ s^-1^) given between the R pulses (Fig 3D). These data suggest that phyB mediates regulation of chlorophyll content through LFR in rice seedlings.

### Chloroplast development is restricted in seedlings displaying pale-green phenotype

**Fig 4:**
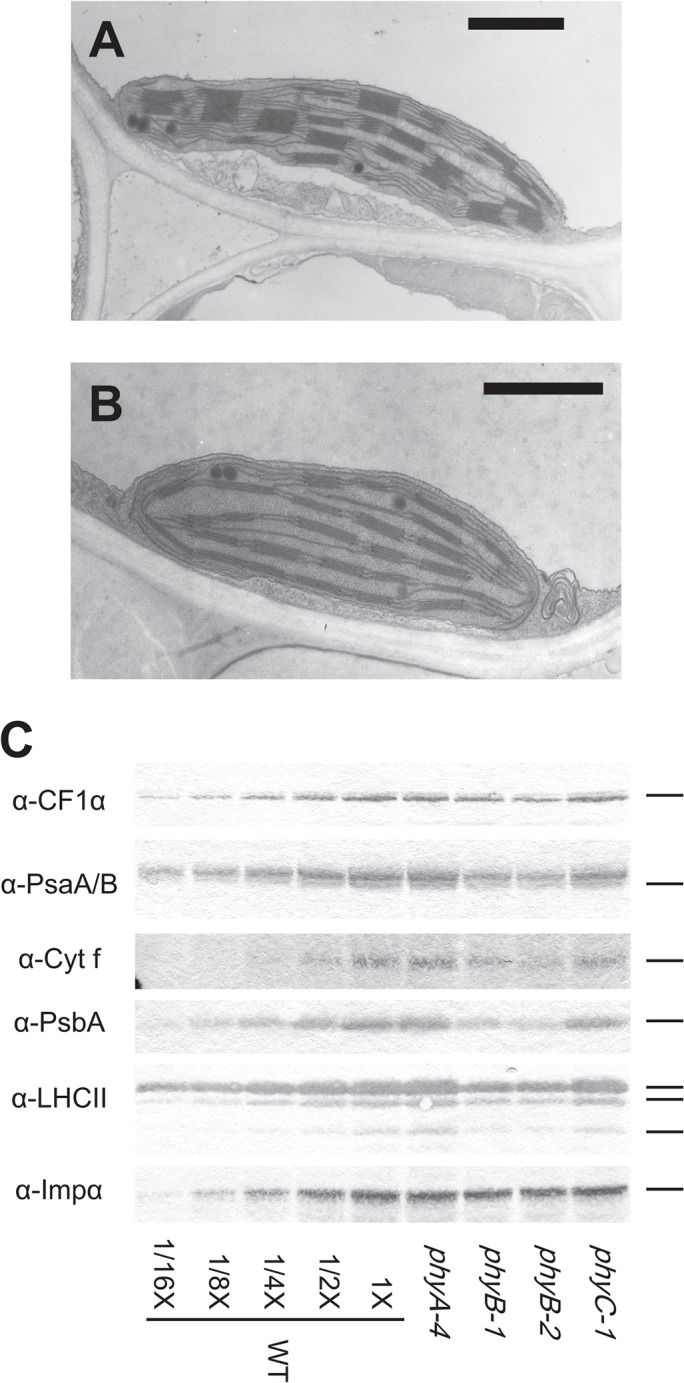
Chloroplast structures and western blots of photosynthesis-related proteins in seedlings grown under Rc irradiation. TEM images showing typical structures of chloroplasts in leaf sheath cells of the WT (A) and *phyB* (B) seedlings grown under Rc (15μmol m^-2^ s^-1^) for 9 days. Scale bars represent 1μm. (C) Western blotting analyses of photosynthesis-related proteins in phytochrome mutants grown under Rc for 9 days using rabbit polyclonal antiserum against CF1α, PsaA/B, Cyt*f*, PsbA and LHCII. Rabbit polyclonal antiserum against rice importin-α (Imp α), a typical housekeeping protein, was used as the loading control. Proteins extracted from 0.67 mg of fresh weight of seedlings were used for this analysis. Dilution series of the WT samples are also shown on the blots. Reproducibility of the result was confirmed with two more biological replicates.

Fig 4A and 4B show ultrastructure of representative plastids in leaf sheath cells of WT and *phyB* seedlings, respectively. The *phyB* seedlings grown under Rc irradiation had chloroplasts (Fig 4B), but development of their thylakoid was poor and their grana stacks were thinner than those of WT (Fig 4A). In addition, chloroplasts in the mutant were substantially small, indicating that development, as well as expansion of chloroplasts, is restricted in the *phyB* mutants. In parallel, levels of photosynthesis-related proteins showed significant decreases in the *phyB* mutants (Fig 4C), which is consistent with the TEM images. CF1α (α subunit of chloroplast coupling factor 1) displayed a slight reduction, while PsaA/B (the reaction center proteins of photosystem I), Cyt *f* (a subunit of cytochrome *b*_*6*_ *f* complex), PsbA (D1 protein, the reaction center protein of photosystem II) and LHCII (light-harvesting complex of photosystem II) showed drastic decrease in their levels in the *phyB* mutants (Fig 4C). Severe reduction of LHCII proteins is likely to be associated with heightened chlorophyll *a*/*b* ratios in the *phyB* mutants (Fig 1C), because the LHCII proteins are known to be the major components available to bind chlorophyll *b* in higher plants [40].

### Pale-green phenotype in *phyB* mutant could be observed in Rc-induced greening process

**Fig 5:**
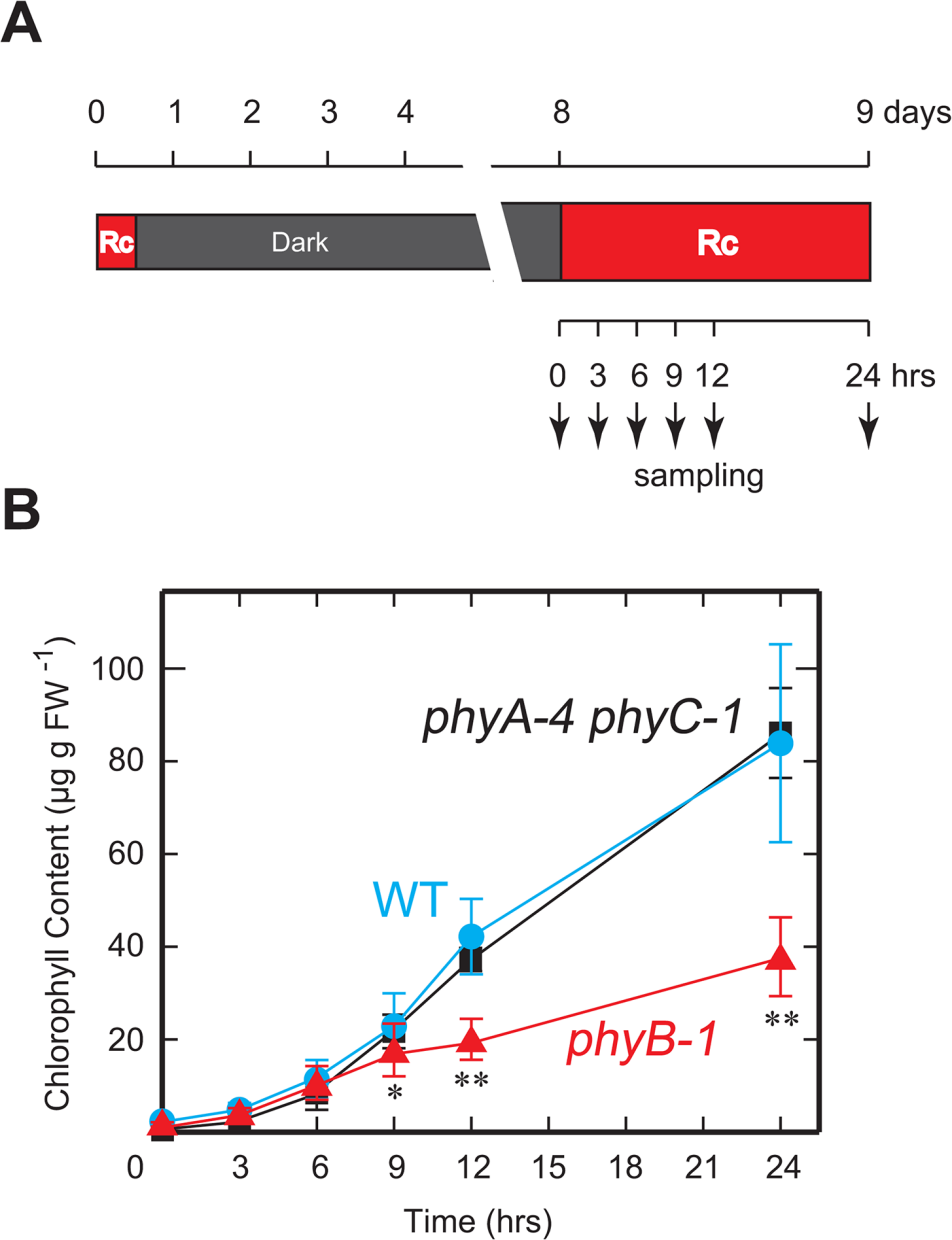
Rc-induced greening experiment. (A) Time line and light regime of the experiment. (B) Kinetics of increase of chlorophyll content in seedlings. The data show means of chlorophyll content from at least 10 biological replicates with SD. Symbols, ** and * indicate statistically significant differences compared to WT at *P* < 0.01 and *P* < 0.05, respectively, calculated by Welch’s *t*-test.

To evaluate the involvement of phytochromes in chlorophyll accumulation during the greening process, we measured chlorophyll content of 8-day-old dark grown seedlings exposed to Rc (15μmol m^-2^ s^-1^) for 24 h and examined the kinetics of chlorophyll accumulation. In this experiment, the seedlings were exposed to Rc twice (Fig 5A). The second irradiation was given to induce chlorophyll accumulation of the seedlings, while the first irradiation was supplied for the first 16 h of cultivation to prevent the appearance of an undesired phenotype (closed coleoptiles with no emergence of leaves) observed in the dark-grown seedlings [15], which hampered the assessments of the chlorophyll accumulation of the leaf sheaths and blades. The first irradiation induced partial photomorphogenesis leading to the emergence of leaves from coleoptiles. We have confirmed that the first irradiation did not induce any chlorophyll accumulation in the 8-day-old dark grown seedlings (Fig 5B).

When 8-day-old dark-grown seedlings were exposed to Rc irradiation, the WT seedlings showed a rapid increase in chlorophyll content after 6 h of moderate increase (Fig 5B). Chlorophyll accumulation in the *phyA phyC* seedlings followed a remarkably similar path as that of the WT seedlings. Despite the initial similarity in the accumulation pattern, a rapid accumulation of chlorophyll did not occur after 6 hours in the *phyB* seedlings after the initial moderate increase (Fig 5B).

### Transcript levels of enzymes on chlorophyll/heme common pathway are comparable between WT and *phyB* seedlings

**Fig 6:**
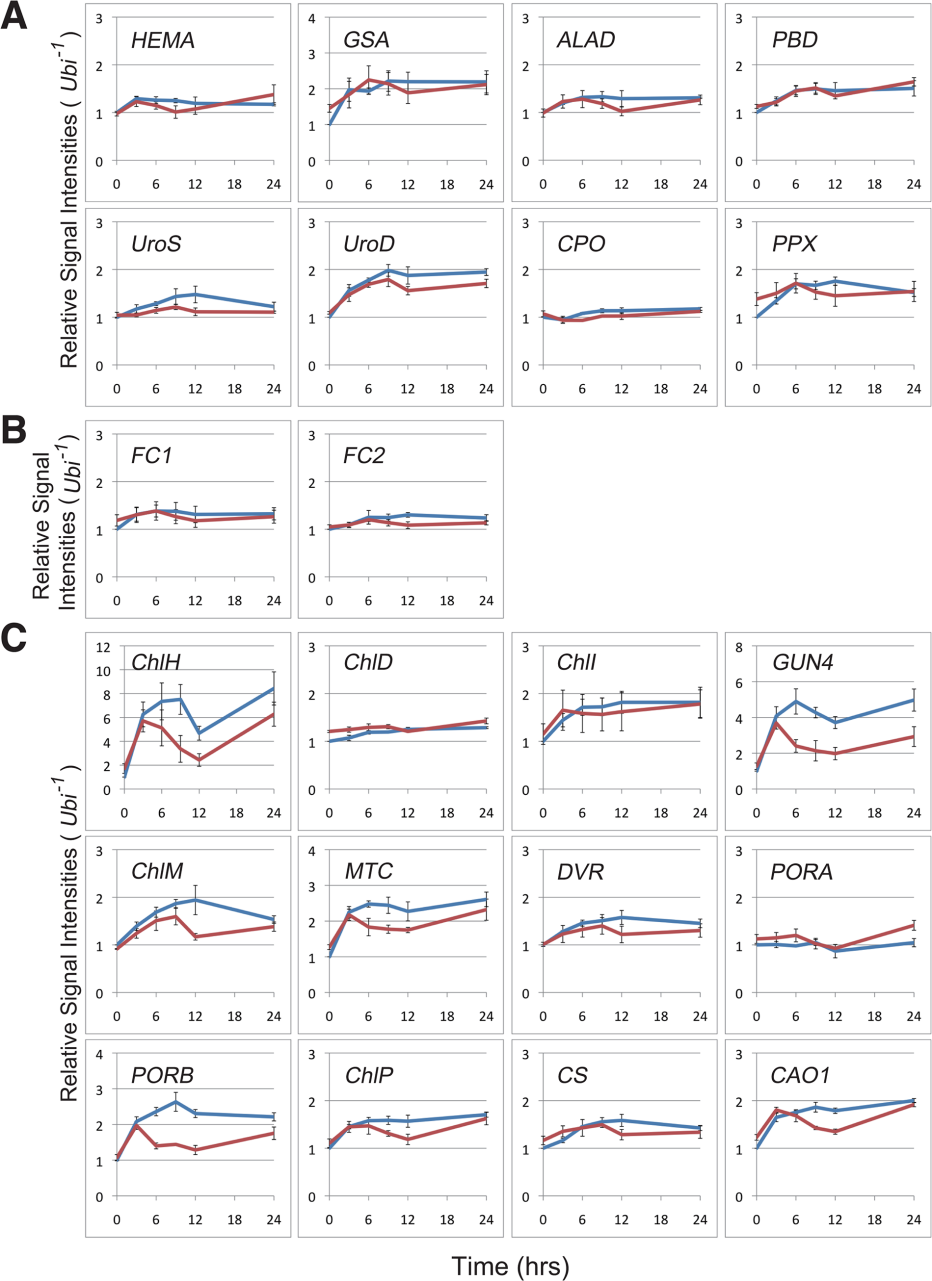
Transcript levels of chlorophll/heme biosynthesis related genes and their changes during the Rc-induced greening experiment. Blue and red lines indicate relative transcript levels of WT and *phyB* seedlings, respectively, which were estimated from ethidium bromide-stained gel images. Each value represents the mean with standard errors of at least three biological replicates. Expression patterns of enzymes involved in the chlorophyll/heme common pathway (A), heme branch (B) and chlorophyll branch (C) are indicated.

The Rc-induced greening experiment revealed that the pale-green phenotype of the *phyB* mutants appeared at an early stage of the greening process (Fig 5B). To better understand the initial event causing the phenotype, we examined the transcript levels of chlorophyll/heme biosynthesis related proteins (S1 Fig, S1 Table) and the changes in transcript levels observed during the Rc-induced greening process (Fig 6). Representative gel images of the RT-PCR analyses are shown in S2 Fig.

Fig 6A shows expression patterns of enzymes involved in the chlorophyll/heme common pathway. We detected an almost two-fold increase in *GSA* transcript levels in the WT seedlings, and the expression in the *phyB* mutant followed a similar path as that of the WT seedlings (Fig 6A). In the case of *UroD* expression, we also observed an almost two-fold increase under Rc in the WT seedlings, but the magnitude of the increase in the mutants was slightly lesser compared to that in the WT. Similar but weaker tendency could be observed in the *UroS* expression levels. On the other hand, the expression level of the remaining enzymes in the chlorophyll/heme common pathway (*HEMA*, *ALAD*, *PBD*, *CPO*, and *PPX*) was almost stable throughout the time course of sampling (up to 24 hours). In addition, the expression levels of these were comparable between the WT and *phyB* seedlings. It is certainly that differences between the WT and *phyB* seedlings were detected in the two exceptions (*UroS* and *UroD*), but the differences could be interpreted to be exceedingly small. These results suggest that a transcriptional regulation of enzymes in the chlorophyll/heme common pathway could not have contributed to the pale green phenotype we observed.

Similar to most of the enzymes in the chlorphyll/heme common pathway, enzymes of the heme branch, *FC1* and *FC2*, were constitutively expressed in both WT and *phyB* seedlings (Fig 6B). In addition, there was no difference in expression patterns between WT and *phyB* mutants of the enzymes involved in the heme branch either.

### phyB is indispensable for the robust expression of some enzymes in the chlorophyll branch

In contrast, we found that the expression levels of some genes in the chlorophyll branch (*ChlH*, *GUN4*, *ChlM*, *MTC* and *PORB*) were clearly induced by Rc (Fig 6C). However, not all genes in the chlorophyll branch exhibited Rc induction. Several of them displayed either a weak induction by Rc (*ChlI*, *DVR*, *ChlP*, *CS* and *CAO1*) or were constitutively expressed (*ChlD* and *PORA*) (Fig 6C). *CAO2* could not be detected under the conditions used in this experiment.

**Fig 7:**
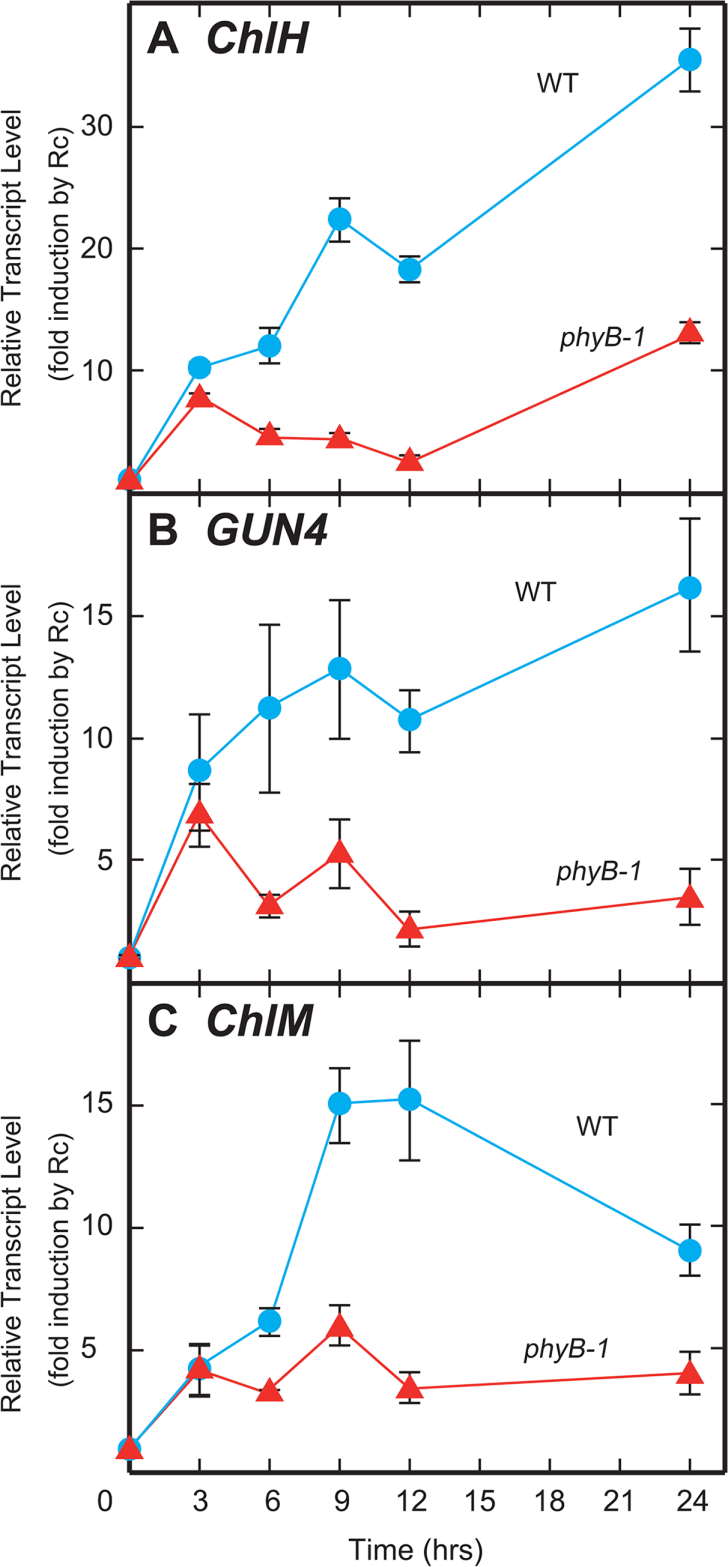
Transcript levels for *ChlH* (A), *GUN4* (B) and *ChlM* (C) during the Rc-induced greening experiment. Each point was plotted at the mean with SD of three replicates, which were estimated by real-time PCR assays. Reproducibility was confirmed with more than two biological replicates.

Interestingly, in all the cases where there was a clear Rc induction, we found significantly elevated levels of expression in WT compared to the *phyB* mutants (Fig 6C), suggesting that there is some sort of a repression in the *phyB* mutants. Among the chlorphyll branch enzymes, *ChlH* encodes the H subunit of Mg-chelatase and *ChlM* encodes Mg-protoporphyrin IX methyltransferase, which are the first and second committing enzymes on the chlorophyll branch (S1 Fig) [20, 21]. GUN4 is known to be an activation factor for Mg-chelatase [23]. These facts imply that the repression of the three genes should have a larger impact on the biosynthesis of chlorophyll in these seedlings. Repression of genes downstream of these in the chlorophyll branch, on the other hand, should not have that great an impact on chlorophyll biosynthesis. Therefore, we focused on *ChlH*, *GUN4* and *ChlM* expression and evaluated the transcript levels of these three genes using a more quantitative procedure, such as real-time PCR. Real-time PCR data obtained for expression levels of *ChlH*, *GUN4*, and *ChlM* corroborated the induction of these genes due to Rc (Fig 7). We observed a 30-fold induction of *ChlH* (Fig 7A) and 15-fold induction of (*GUN4* and *ChlM* (Fig 7B and 7C) within 24 h of irradiation in the WT. A similar, but attenuated induction was observed in the *phyB* seedlings as well (10-fold (*ChlH*), 7-fold (*GUN4*) and 5-fold (*ChlM*)) (Fig 7).

### Mg-protoporphyrin IX levels displayed reduction in parallel with *ChlH* and *GUN4* transcripts levels

**Fig 8:**
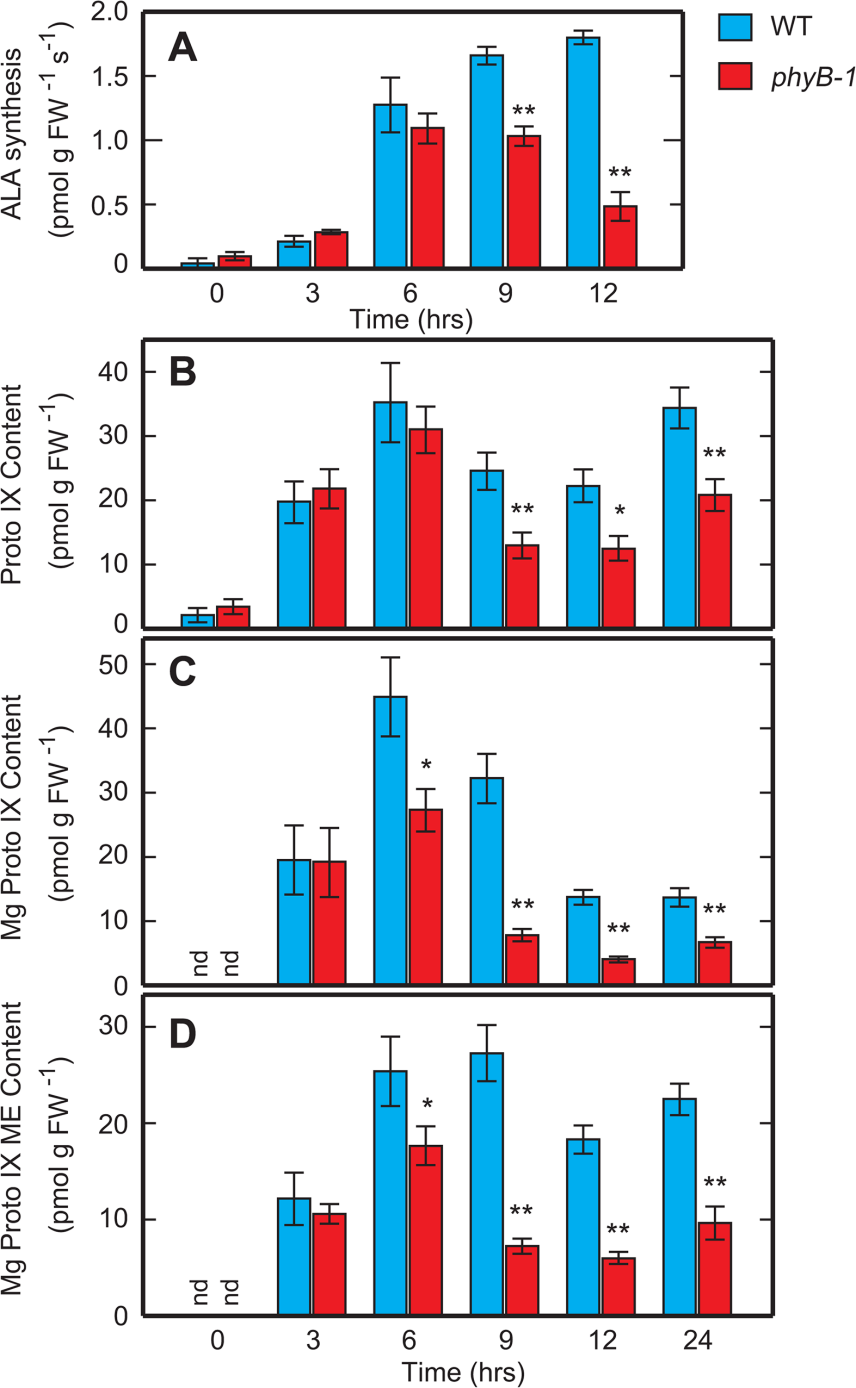
Content of chlorophyll/heme precursors and their changes during the Rc-induced greening process. Rate of 5-ALA biosynthesis (A) and content of the chlorophyll precursors, protoporphyrin IX (B), Mg-protoporphyrin IX (C) and Mg-protoporphyrin IX monomethylester (D), in the WT and *phyB* seedlings and the changes during the Rc-induced greening experiment. Values of 5-ALA biosynthesis rates (A) were estimated from the accumulation of 5-ALA during incubation with levulinic acid for 90 min. Each value represents the mean with standard errors of at least six replicates. Symbols, ** and * indicate statistically significant differences compared to WT at a levels of *P* < 0.01 and *P* < 0.05, respectively, calculated by Welch’s *t*-test. nd, not detected.

We rationalized that a repressed induction in *ChlH*, *GUN4* and *ChlM* genes might lead to the reduction in the activity of the downstream enzymes Mg-chelatase and Mg-protoporphyrin IX methyltransferase. To test this hypothesis, we examined the metabolic flux around these enzymes by measuring the rates of 5-ALA biosynthesis (Fig 8A) and the endogenous content of the three downstream chlorophyll precursors, protoporphyrin IX (Fig 8B), Mg-protoporphyrin IX (Fig 8C) and Mg-protoporphyrin IX monomethylester (Fig 8D) during the Rc-induced greening process. Both the synthesis of 5-ALA, as well as the content of all the downstream precursors, were extremely low in the beginning of our measurements (at time 0) (Fig 8A, 8B, 8C and 8D). With the progression of Rc irradiation, a steady activation of the pathway could be observed. In the WT seedlings, the rate of 5-ALA synthesis gradually increased for the first 6 h of irradiation and eventually reached a plateau (Fig 8A). Similarly, the levels of protoporphyrin IX and Mg-protoporphyrin IX monomethylester also rose rapidly and reached a plateau after 6 h of Rc irradiation (Fig 8B and 8D). Levels of Mg-protoporphyrin IX displayed a slightly different pattern, forming a transient peak at 6 h after Rc irradiation, followed by a decline that stopped at approximately 30% of the peak value (Fig 8C). In the *phyB* mutant, acute increase in Mg-protoporphyrin IX and Mg-protoporphyrin IX monomethylester levels could be observed with kinetics similar to the WT’s in the first few hours. However, the magnitude of increase in the mutant was lesser compared to the WTs, with the initial rapid increase losing momentum after the first 3 h of Rc irradiation (Fig 8C and 8D). Subsequently, the mutant plants displayed different kinetics in that, after the initial increase with a subdued momentum, there was a precipitous drop in Mg-protoporphyrin IX and Mg-protoporphyrin IX monomethylester levels. The levels in the mutant were significantly different from that of the WT from 6 hours onwards (Fig 8C and 8D). The levels of Mg-protoporphyrin IX in the mutant at 6 h of Rc irradiation were approximately 60% of that of WT. At this time point, the level of protoporphyrin IX in the mutant was approximately 30 pmol g FW^-1^ and this value was comparable to that of the WT seedlings, which suggests that the substrate of Mg-chelatase was not a limiting factor. However the product of this enzyme, Mg-protoporphyrin IX, was significantly reduced, suggesting that the Mg-chelatase activity was lowered in the mutant at that time. It is noteworthy to mention that the kinetics of the reduction of Mg-protoporphyrin IX levels parallels the reduction of transcripts for *ChlH* and *GUN4* (Fig 7A and 7B). We also detected a decline of Mg-protoporphyrin IX monomethylester levels in the mutant (Fig 8D). However, it is difficult to evaluate whether or not this decline is linked with the reduced activity of the Mg-protoporphyrin IX methyltransferase. Mg-protoporphyrin IX, the substrate of Mg-protoporphyrin IX methyltransferase, has shown a parallel decline with Mg-protoporphyrin IX monomethylester’s in our experiments, indicating a simultaneous substrate limitation in this case.

5-ALA biosynthesis rate and protoporphyrin IX content were reduced in the mutant. Both reductions appeared after 9 h of Rc irradiation, indicating that they were clearly posterior events to the reductions of Mg-protoporphyrin IX and Mg-protoporphyrin IX monomethylester content (Fig 8A, 8B, 8C and 8D).

## Discussion

In angiosperms, dark-grown seedlings display skotomorphogenesis; they grow heterotrophically and depend on the stored material in their seeds. However, once the seedlings perceive light signals, a dynamic transition to photomorphogenesis is triggered for a rapid shift to photoautotrophic growth [30]. In this shift, efficient chloroplast development is thought to be crucial. In the initial phase of chloroplast development, chlorophyll synthesis is massively activated to respond to the demand for building up the photosynthetic machinery. However, chlorophyll synthesis must be strictly regulated at this phase, because insufficient amounts of chlorophylls will retard chloroplast development, while excessive amounts of chlorophyll and its tetrapyrrole precursors will increase the risk of photo-oxidative damage to the developing chloroplasts [21]. Therefore, angiosperms should be equipped with several layers of regulatory mechanisms of chlorophyll synthesis during the greening process. In this study, we focused on the phyB-mediated regulation of chlorophyll synthesis in rice seedlings. To elucidate this regulation, we probed the molecular basis of the pale-green phenotype observed in the mutant *phyB* seedlings grown under Rc irradiation.

### phyB signal is crucial for robust chlorophyll accumulation and chloroplast development

*phyB* seedlings grown under Rc irradiation displayed a pale-green phenotype due to the reduction of chlorophyll content, while deficiency in other phytochromes did not diminish the chlorophyll levels significantly. These results reveal that chlorophyll accumulation by Rc irradiation can be divided into two components—the phyB-dependent and the phyB-independent ones. In the *phyB* seedlings, the phyB-independent component is still active, allowing low accumulation of chlorophyll and yielding the pale-green phenotype of the mutant. The phenotype can be mimicked even in the WT seedlings by treatments that are able to cancel induced LFRs (Fig 3B and 3D). It is logical that the phyB-dependent component of chlorophyll accumulation could be diminished by such treatments since phyB is known to be a major photoreceptor mediating the LFRs in many plant species [10]. Our results indicate that phyB is crucial for robust chlorophyll accumulation under Rc irradiation and the phyB-mediated regulation is based on the mode of LFR.

Chloroplasts could be observed in the *phyB* seedlings displaying the pale-green phenotype, but their development was limited. Amounts of thylakoid proteins were also diminished in the mutant in parallel with the level of thylakoid development in the chloroplasts, implying that the components of the thylakoid were severely altered. However almost normal photosynthetic electron transport activities could be detected in thylakoid isolated from the *phyB* mutants when the activities were calculated on a chlorophyll basis (S2 Table). These observations indicated that phyB deficiency did not affect photosystems themselves, but amounts of the photosystems on the basis of fresh weight were significantly reduced. It implies that phyB deficiency does not block a critical process in chloroplast development. It is more likely that phyB deficiency restricts the supply of an important component, probably chlorophylls, which limits chloroplast development in the mutant. Similar results have been obtained from *Arabidopsis* plants as well. When *Arabidopsis* plants face limitations in chlorophyll supplies, they overcome the shortage through repression of chloroplast development. The *Arabidopsis* mutant *chl27*, in which expression of the Mg-protoporphyrin IX monomethylester cyclase gene is repressed by a T-DNA insertion in its promoter region, shows retarded growth and a severe pale green phenotype. Interestingly, the mutant has chloroplasts with less-developed thylakoid membranes. In addition, the integrity of each photosystem was also shown to be almost normal [41]. Our results suggest that rice plants are also equipped with similar mechanisms to align chloroplast development with utilizable amounts of chlorophylls. Molecular mechanisms of the alignment remain to be elucidated. It is probable that the reduced chlorophyll synthesis in the chloroplasts would lead to suppression of nuclear gene expression required for construction of photosynthesis machineries via retrograde signaling [42].

To evaluate the hypothesis that phyB deficiency restricts the supply of chlorophylls under Rc irradiation, we established an Rc-induced greening experiment and examined the early phase of chlorophyll accumulation in the *phyB* mutant. Results of the experiment indicated that Rc-induced chlorophyll accumulation could be divided into two components based on phyB-dependency. The phyB-independent component was observed as a rapid, but moderate induction, while the phyB-dependent part appeared after 9 h of Rc irradiation as a brisk increase in chlorophyll content (Fig 5B). The pale-green phenotype of the *phyB* mutant could, therefore, be attributed to the loss of the phyB-dependent component of chlorophyll accumulation. We also examined phyB-independent component in the early phase of chlorophyll accumulation. Surprisingly, this rapid, but moderate induction could be detected in the *phyA phyC* seedlings despite the fact that the mutant has only phyB as a photoactive phytochrome in the cells. These results suggest that the phyB-independent activities detected in the initial phase of the greening process are attributed to the autonomous photoconversion of protochlorophyllides [28], which massively accumulate in dark-grown seedlings [29].

### Mg-chelatase activity is regulated by phyB-mediated signaling through transcriptional regulation

To elucidate the molecular basis of the loss of phyB-dependent chlorophyll accumulation in the *phyB* mutant, we assessed the expression of the genes involved in chlorophyll synthesis. Our analyses revealed that considerable numbers of transcripts encoding proteins acting on the chlorophyll branch were significantly repressed in their levels in the mutant. Especially, *ChlH* and *GUN4* showed clear Rc-induction in the WT seedlings and their induction was reduced to almost one-third in the *phyB* mutants. Similar induction by Rc of the orthologues of *ChlH* and *GUN4* in *Arabidopsis*, At *ChlH* and At *GUN4*, have been shown by Stephenson and Terry [27] during the Rc-induced greening (de-etiolation) process. Similar to our results, these inductions were also diminished in the *Arabidopsis phyB* mutant [27]. These observations suggest that *ChlH* and *GUN4* are regulated by common mechanisms in rice and *Arabidopsis*.

Previous results have shown that the expression of genes encoding proteins acting on the chlorophyll branch might be controlled by the *GLK1* (Golden2-like 1) gene [43]. Overexpression of *GLK1* in rice calli resulted in massive induction of *ChlH*, *ChlM*, *Chl27* (= *MTC* in this study), *PORA*, *PORB* and *ChlP* genes [43]. Almost all of the genes displayed not only clear Rc induction in our experiment but also a significant repression in the *phyB* seedlings. Interestingly, the overexpression of *GLK1* only slightly affects the expression of the genes acting on the chlorophyll/heme common pathway [43], which is also consistent with our observation that these genes were not repressed in the *phyB* seedlings in our experiment (Fig 6).

We next considered how the repressed expression of these genes affects chlorophyll synthesis in the *phyB* mutant. Rc-induction of these transcripts could also be subdivided into two components, a phyB-dependent one and a phyB-independent one. Similar to the kinetics of chlorophyll accumulation, the phyB-independent induction was rapid, but moderate, while the phyB-dependent accumulation appeared only after 6 h of Rc irradiation, but was stronger. Since *ChlH* and *GUN4* encode the catalytic subunit of the Mg-chelatase and its activating protein, respectively, the absence of the phyB-dependent induction of these genes should lead to a decline in Mg-chelatase activity in the *phyB* mutant, especially, after 6 h of Rc irradiation. As expected, the level of Mg-protoporphyrin IX, a product of the Mg-chelatase, was significantly reduced at 6 h of Rc irradiation in the *phyB* mutant. On the other hand, protoporphyrin IX, the substrate of Mg-chelatase, could be detected almost at a comparable level in the mutant at the same time, implying that the substrate level was not a limiting factor. Protoporphyrin IX reduction was observed in our experiment after 9 h of Rc irradiation (Fig 8B), which is at a stage even posterior to the reduction of Mg-protoporphyrin IX content, suggesting that it is a secondary event. Our results suggest a model in which the perception of R by phyB triggers a cascade of expression of genes encoding proteins acting on the chlorophyll branch, including *ChlH* and *GUN4*. Induction of these genes leads to the activation of chlorophyll synthesis through the expression of biosynthetic enzymes, including Mg-chelatase. As just described, the phyB-dependent activation of chlorophyll synthesis should contribute partly to respond to the acute demand of chlorophylls in the greening process.

### Repression of 5-ALA formation is important to stabilize the pale-green phenotype

Reduction of Mg-chelatase activity in the *phyB* seedlings should lead to the accumulation of protoporphyrin IX, which is known to be a hazardous photosensitive product. Plants have developed several mechanisms for preventing the accumulation of these tetrapyrrole products and for protecting themselves against this risk. This idea was suggested by researches using transgenic *Arabidopsis* plants in which Mg-chelatase activity was reduced by the artificial suppression of *ChlH* or *ChlI* expression. [44, 45]. These plants displayed a severe pale-green phenotype, but never showed necrosis, which implies that they can manage levels of such hazardous products within permissive limits. As expected, 5-ALA formation rate was significantly reduced in these plants [44, 45], which should prevent excess accumulation of protoporphyrin IX. Similar phenomena could be observed in rice plants as well. The rice Mg-chelatase deficient mutant displayed an albino phenotype, but never showed any necrotic phenotype [46]. In our experiment, we detected a reduction of 5-ALA synthesis in the *phyB* mutant after a reduction of Mg-chelatase activity (Fig 8). These observations imply that rice plants also have a similar protective mechanism against a situation in which chlorophyll synthesis is restricted at the level of Mg-chelatase reaction. Several models of post-translational regulation have been reported for managing hazardous products in plants. Terry and Kendrick proposed that, in *Arabidopsis* plants, excessive amount of heme acts as a signaling component to repress the activity of glutamyl-tRNA reductase post-translationally [47]. The existence of the GUN4-mediated post-translational mechanism to repress glutamyl-tRNA reductase has also been reported [48]. The molecular mechanism to manage hazardous products in the rice *phyB* mutant is still unclear, but our data indicate that the reduction of Mg-chelatase activity in the mutant consequently leads to the repression of 5-ALA formation, which probably stabilizes the pale-green phenotype without any accumulation of hazardous chlorophyll precursors.

### phyB-mediated regulation of chlorophyll synthesis might contribute to the repression of chloroplast development in shaded tissue

Results of our experiments revealed several features of phyB-mediated and LFR-based chlorophyll synthesis regulation in rice seedlings. We further considered the physiological meaning of this regulation in field-grown plants. The induction of the pale-green phenotype in the WT seedlings by FR-enriched light suggests that this phenotype is one of the shade avoidance responses in rice plants [49]. Repression of chloroplast development is thought to be advantageous in shaded tissues because their chloroplasts cannot contribute enough photosynthetic assimilation due to limitations in the availability of light energy. If plants save energy by repressing chloroplast development in such conditions, they can utilize the conserved energy for other pressing responses. To confirm the interpretation, further studies using adult plants will be absolutely needed.

## Supporting Information

S1 Fig Chlorophyll/heme biosynthesis pathway in plants. Each arrow indicates a step of the reaction catalyzed by an enzyme. Pivotal precursors and products in the pathway are indicated in bold. Genes analyzed in this study are shown in italics. Further information on these genes is summarized in S1 Table. Abbreviations: Glu-tRNA, glutamyl-tRNA; Glu TR, glutamyl-tRNA reductase; 5-ALA, 5-aminolevulinic acid; Proto IX, protoporphyrin IX; MgCh, Mg-chelatase; Mg-Proto IX, Mg-protoporphyrin IX; MgMT, Mg protoporphyrin IX methyltransferase; Mg-Proto IX ME, Mg-protoporphyrin IX monomethylester; PChlide, protochlorophyllide; Chlide, chlorophyllide; Chl, chlorophyll.
(PDF)

S2 Fig RT-PCR to detect transcripts involved in chlorophyll/heme biosynthesis pathway. Representative gel images of RT-PCR for transcripts involved in chlorophyll/heme common pathway (A), heme branch (B) and chlorophyll branch (C) during the Rc-induced greening experiment. Numbers on right side of the columns represent cycles applied in PCR amplification.
(PDF)

S1 Table Genes examined and PCR primer sequences used to amplify their transcripts. (PDF)

S2 Table Electron transport activities of photosystem I and II in thylakoid membranes isolated from the WT and *phyB-1* seedlings grown under Rc irradiation for 9 days. (PDF)
