# Supplementary material for: Phytochrome B Mediates the Regulation of Chlorophyll Biosynthesis through Transcriptional Regulation of ChlH and GUN4 in Rice Seedlings
Source: PLoS One. 2015 Aug 13;10(8):e0135408. doi: 10.1371/journal.pone.0135408 (PMC4536196; doi:10.1371/journal.pone.0135408)
Supplement: S1 Fig — Each arrow indicates a step of the reaction catalyzed by an enzyme. Pivotal precursors and products in the pathway are indicated in bold. Genes analyzed in this study are shown in italics. Further information on these genes is summarized in S1 Table. Abbreviations: Glu-tRNA, glutamyl-tRNA; Glu TR, glutamyl-tRNA reductase; 5-ALA, 5-aminolevulinic acid; Proto IX, protoporphyrin IX; MgCh, Mg-chelatase; Mg-Proto IX, Mg-protoporphyrin IX; MgMT, Mg protoporphyrin IX methyltransferase; Mg-Proto IX ME, Mg-protoporphyrin IX monomethylester; PChlide, protochlorophyllide; Chlide, chlorophyllide; Chl, chlorophyll. (PDF) [file pone.0135408.s001.pdf]

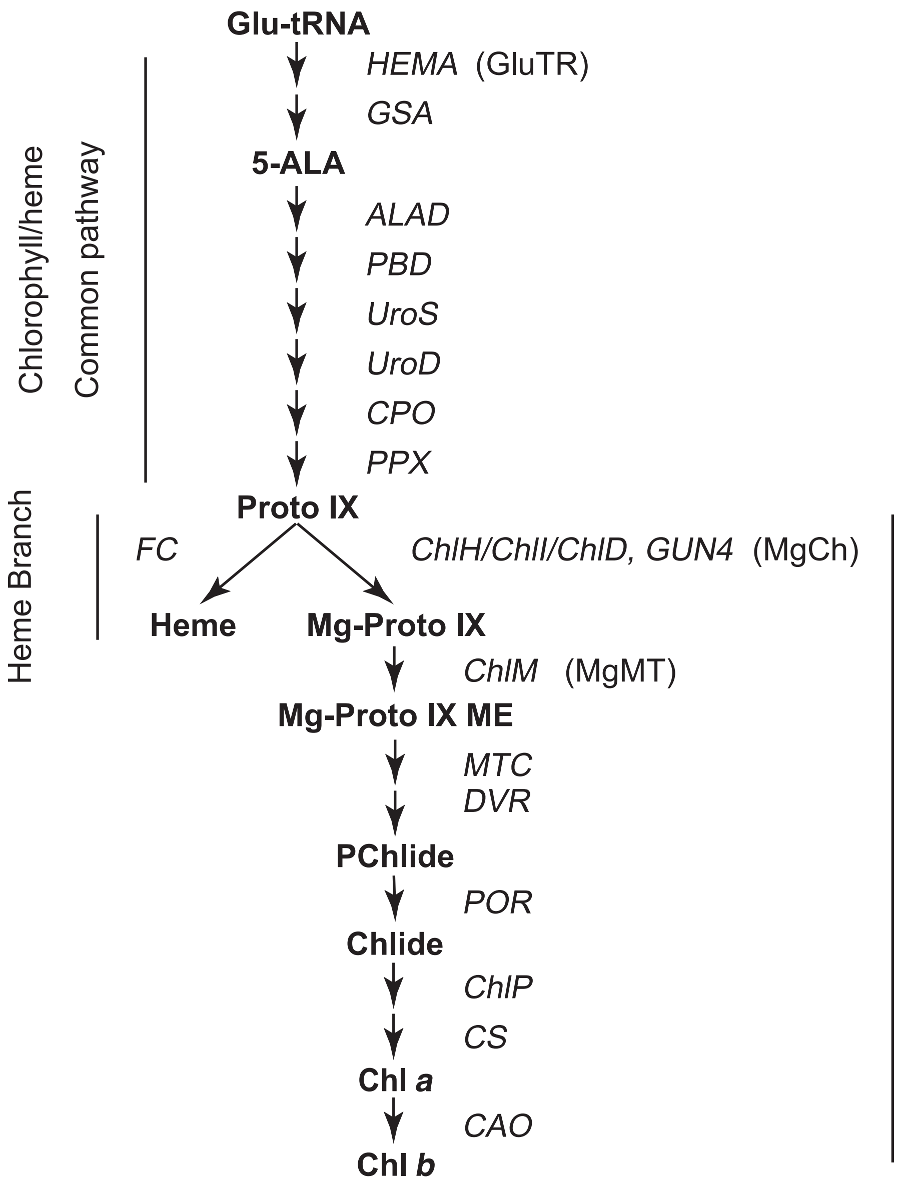

S1 Fig. Chlorophyll/heme biosynthesis pathway in plants.

Each arrow indicates a step of the reaction catalyzed by an enzyme. Pivotal precursors and products in the pathway are indicated in bold. Genes analyzed in this study are shown in italics. Further information on these genes is summarized in S1 Table. Abbreviations: Glu-tRNA, glutamyl-tRNA; Glu TR, glutamyl-tRNA reductase; 5-ALA, 5-aminolevulinic acid; Proto IX, protoporphyrin IX; MgCh, Mg-chelatase; Mg-Proto IX, Mg-protoporphyrin IX; MgMT, Mg protoporphyrin IX methyltransferase; Mg-Proto IX ME, Mg-protoporphyrin IX monomethylester; PChlide, protochlorophyllide; Chlide, chlorophyllide; Chl, chlorophyll.
