# Supplementary material for: Phytochrome B Mediates the Regulation of Chlorophyll Biosynthesis through Transcriptional Regulation of ChlH and GUN4 in Rice Seedlings
Source: PLoS One. 2015 Aug 13;10(8):e0135408. doi: 10.1371/journal.pone.0135408 (PMC4536196; doi:10.1371/journal.pone.0135408)
Supplement: S2 Fig — Representative gel images of RT-PCR for transcripts involved in chlorophyll/heme common pathway (A), heme branch (B) and chlorophyll branch (C) during the Rc-induced greening experiment. Numbers on right side of the columns represent cycles applied in PCR amplification. (PDF) [file pone.0135408.s002.pdf]

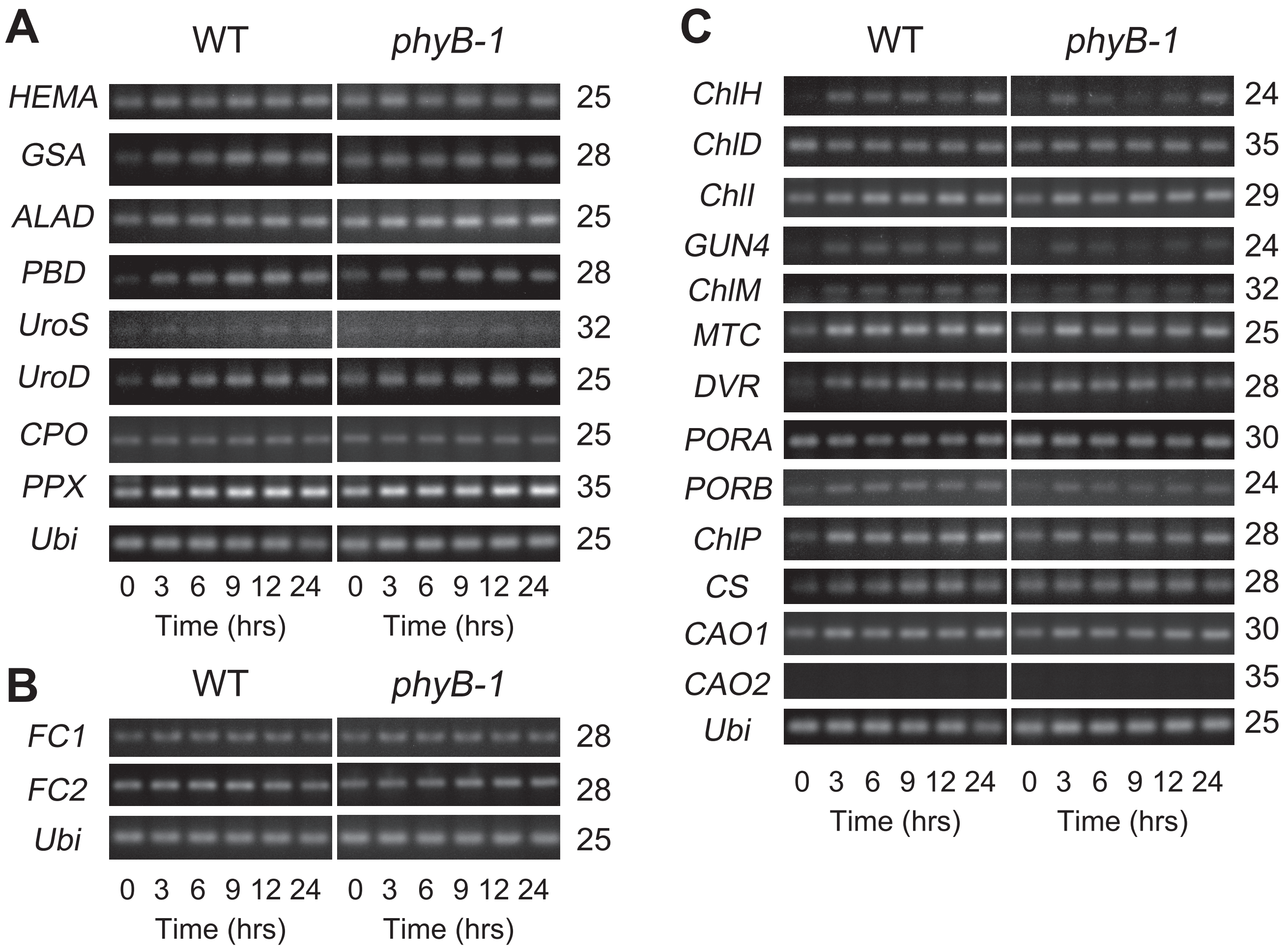

S2 Fig. RT-PCR to detect transcripts involved in chlorophyll/heme biosynthesis pathway. Representative gel images of RT-PCR for transcripts involved in chlorophyll/heme common pathway (A), heme branch (B) and chlorophyll branch (C) during the Rc-induced greening experiment. Numbers on right side of the columns represent cycles applied in PCR amplification.

N. Inagaki et al.
