# Supplementary material for: Phytochrome B Mediates the Regulation of Chlorophyll Biosynthesis through Transcriptional Regulation of ChlH and GUN4 in Rice Seedlings
Source: PLoS One. 2015 Aug 13;10(8):e0135408. doi: 10.1371/journal.pone.0135408 (PMC4536196; doi:10.1371/journal.pone.0135408)
Supplement: S1 Table — (PDF) [file pone.0135408.s003.pdf]

S1 Table. Genes examined and PCR primer sequences used to amplify their transcripts.

Symbol	Common name	RAP ID	FW primer	RV primer	Ref.
Chlorophyll/heme common pathway					
HEMA	Glutamyl-tRNA reductase (GluTR)	Os10g0502400	5'-CGTCGATGACTTGAAAGAGGT-3'	5'-GATTGTTTGGGCCTCCATT-3'	1
GSA	Glutamate-1-semialdehyde 2,1-aminomutase	Os08g0532200	5'-GAAGTCCGAGGAGATCTTCAAC-3'	5'-GAAGGCACGAACTGGTGAAT-3'	1
ALAD	5-aminolevulinate dehydrogenase	Os06g0704600	5'-ATTGCCAATCGCTGCATATC-3'	5'-CCATCATCACCTTCTCTTCGT-3'	1
PBD	Porphobilinogen deaminase	Os02g0168800	5'-GCCAAGGTCTCGCTCATC-3'	5'-CCCTCCTCGGCTAACTCTG-3'	1
UroS	Uroporphyrinogen-III synthase	Os03g0186100	5'-GAGGTTCATAGCGTGCTCCT-3'	5'-CAAGACTTTGTACATTATGTTTTTCCA-3'	1
UroD	Uroporphyrinogen-III decarboxylase	Os03g0337600	5'-TGCTGTGAATGAGGTCAGAGA-3'	5'-ACACGGCAGCTTCATTGTTA-3'	1
CPO	Coproporphyrinogen oxidase	Os04g0610800	5'-TTGAGACAGATGCACCGAAA-3'	5'-CACACGCTTGTTTTTGAACAG-3'	1
PPX	Proporphyrinogen oxidase	Os01g0286600	5'-TGGGAGGAGGGGCCCAACAGCTTCC-3'	5'-CCACAGCACGAACCGCGGCGCGTTG-3'	2
Heme branch					
FC1	Ferrochelatase	Os09g0297000	5'-GGTCAACAGGGTGTAAAGAG-3'	5'-GTGCATCCAAGAGCTGGAAC-3'	2
FC2	Ferrochelatase	Os05g0361200	5'-CTTGCCTTATGTTGGTGCTA-3'	5'-AGCCCCACTCCCATACAGTC-3'	2
Chlrophyll branch					
ChlH	Magnesium chelatase (MgCh) H subuint	Os03g0323200	5'-CCAATCCGTAACCCGAAGGT-3'	5'-CAATAATTTTGGCGCTCTTCAA-3'	2
ChlD	MgCh D subuint	Os03g0811100	5'-CCAACGGGGCCATCCCCTCGGGGAA-3'	5'-TTGCCCCAAGCAGCAGAGCAGTTTT-3'	2
ChlI	MgCh I subuint	Os03g0563300	5'-CCCGCCGCGTCGCCGGCCCTCTTCT-3'	5'-CGGCGGAACCCTCTCCGTGACGGGA-3'	2
GUN4	Genome uncoupled 4	Os11g0267000	5'-GCAGCAGAGGAGAATGACACACCTG-3'	5'-GTAGTCAGGCTTGAAGAAATCTCTC-3'	2
ChlM	Mg-Proto IX methyltransferase (MgMT)	Os06g0132400	5'-TCGACGTGCTGATCCACTAC-3'	5'-TGATGAGCACCCGCTTCT-3'	1
MTC	Mg-Proto IX monomethylester cyclase	Os01g0279100	5'-CCGCAGGCTCAAGAAAAC-3'	5'-ATCAGACAGCCCCTTGTTCA-3'	1
DVR	3,8-divinyl protochlorophyllide *a* 8-vinyl reductase	Os03g0351200	5'-CCATTGCCAGTTTCTTGGTG-3'	5'-AATTGAATGGCTAATGGCGT-3'	2
PORA	Protochlorophyllide oxidoreductase (POR)	Os04g0678700	5'-AAGTGGCACGTGGTGATG-3'	5'-AGGTGCATGACGGTGTAGC-3'	1
PORB	Protochlorophyllide oxidoreductase (POR)	Os10g0496900	5'-GATGGAGGAGAATTTGTAGG-3'	5'-ATGATCCACCAGTGAACAAC-3'	2
ChlP	Geranylgeranyl-diphosphate reductase	Os02g0744900	5'-ATCCCCGACGACAAGATG-3'	5'-GTCGCACTTGGGGAACAC-3'	1
CS	Chlorophyll synthase	Os05g0349700	5'-AGGCTCCAGCTTCAACCAG-3'	5'-GGCGAATCTTCCATATGTCG-3'	1
CAO1	Chlorophyll *a* oxygenase	Os10g0567400	5'-TGCTCATCAAGCCTTCCTTCAGGTG-3'	5'-GCGTTGTTCTTTGCATAGTCAGCTC-3'	2
CAO2	Chlorophyll *a* oxygenase	Os10g0567100	5'-CTGCTCGTCAAGCCTTCCTCCTCCT-3'	5'-CCCTCATGTTGTTCTTGGCATGTTG-3'	2
Control					
Ubi	Ubiquitin	Os02g0161900	5'-GAGCCTCTGTTCGTCAAGTA-3'	5'-ACTCGATGGTCCATTAAACC-3'	2

1 H. Nakamura and H. Ichikawa, personal communication

2 in this work
