# Supplementary material for: Phytochrome B Mediates the Regulation of Chlorophyll Biosynthesis through Transcriptional Regulation of ChlH and GUN4 in Rice Seedlings
Source: PLoS One. 2015 Aug 13;10(8):e0135408. doi: 10.1371/journal.pone.0135408 (PMC4536196; doi:10.1371/journal.pone.0135408)
Supplement: S2 Table — (PDF) [file pone.0135408.s004.pdf]

**S2 Table**. **Electron transport activities of photosystem I and II in thylakoid membranes isolated from the WT and *phyB-1* seedlings grown under Rc irradiation for 9 days.**

| Photosystem II activity ($\mu$mol $e^-$ mg $Chl^{-1}$ $h^{-1}$) | | Photosystem I activity ($\mu$mol $e^-$ mg $Chl^{-1}$ $h^{-1}$) | |
|---|---|---|---|
| WT | *phyB-1* | WT | *phyB-1* |
| 487.3±51.1 | 461.9±29.4 | 279.2±14.3 | 242.4±7.6 |

Each value is the mean of data from 4 samples with their SD.

**Procedures**

**Isolation of thylakoid membranes**

Seedlings grown under Rc irradiation for 9 days were harvested, weighed and ground in 8-fold volume of 50 mM Tris HCl buffer (pH 7.5) containing 0.1 M sucrose, 10 mM NaCl, 2.5 mM $MgCl_2$ and 10 mM sodium ascorbate by a Waring blender. The homogenate was filtered through two layers of cheesecloth and centrifuged at 300 ×*g* for 30 sec. The supernatant was further centrifuged at 20,000 ×*g* for 7 min. The precipitate was resuspended with the same buffer and then centrifuged at 20,000 ×*g* for 7 min. Crude thylakoid was obtained as the resultant precipitate, which was suspended into small volume of a measuring buffer, 50 mM Mes-NaOH (pH 6.5) containing 0.1M sucrose, 10 mM NaCl and 5 mM $MgCl_2$.

**Measurement of PSII activity**

Photosynthetic electron transport activity of PSII was estimated from photosynthetic oxygen evolution rate measured by a Clark-type oxygen electrode (Rank Brothers Ltd). Ten μg chlorophyll $ml^{-1}$ of thylakoid in the measuring buffer in a glass chamber with the electrode was irradiated by saturating actinic light from a halogen lamp in the presence of 0.8 mM 2,6-dichlorobenzoquinone (DCBQ) and 1 mM potassium ferricyanide.

**Measurement of PSI activity**

Photosynthetic electron transport activity of PSI was estimated from decrease of oxygen molecules by re-oxidation of photosynthetically reduced methyl viologen (MV) measured by a Clark-type oxygen electrode (Rank Brothers Ltd). Four μg chlorophyll $ml^{-1}$ of thylakoid in the measuring buffer was exposed to a saturating actinic light in the electrode chamber in the presence of 1 mM KCN, 0.07 mM reduced 2,6-dichloroindophenol (DCIP), 0.1 mM MV, 0.01 mM 3-(3,4-Dichlorophenyl)-1,1-demethylurea (DCMU) and 0.01 mM carbonylcyanide-3-chlorophenylhydrazone (CCCP).
